# Lobomycosis: single lesion on the lip^☆^

**DOI:** 10.1016/j.abd.2024.03.004

**Published:** 2024-09-05

**Authors:** Kananda Kesye Sousa Nunes, Carlos Alberto Chirano Rodrigues, Antonio Pedro Mendes Schettini, Sinésio Talhari

**Affiliations:** Department of Dermatology, Fundação Hospitalar Alfredo da Matta, Manaus, AM, Brazil

*Dear Editor,*

A 66-year-old male patient, a farmer from Coari, state of Amazonas, Brazil, had a lesion on the upper lip with a 10-year evolution. On examination, an erythematous, infiltrated tumor lesion with a firm consistency was observed on the right side of the upper lip (Fig. 1). The general clinical examination and laboratory tests showed no changes.

**Figure 1:**
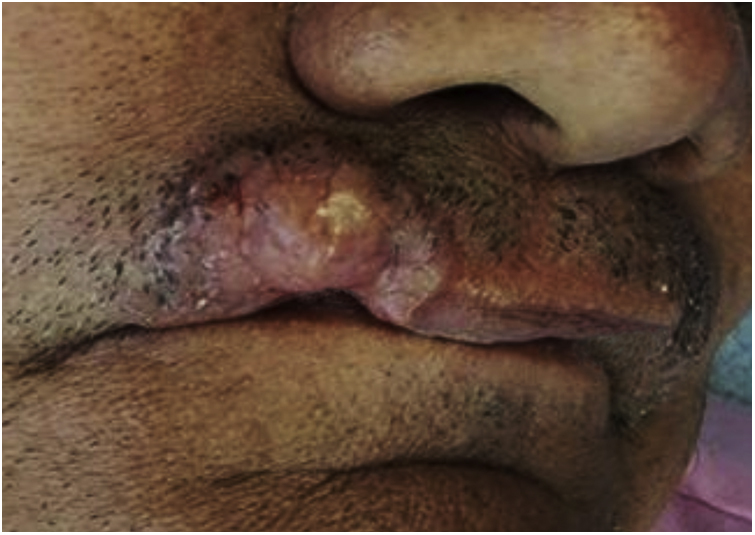
Tumor lesion with a 10-year evolution.

A biopsy of the lesion was performed and histopathology revealed a nodular granulomatous inflammatory infiltrate involving the entire dermis and hypodermis, consisting of epithelioid histiocytes and numerous giant cells, containing rounded fungal structures in a catenulate arrangement compatible with *Lacazia loboi* (Figure 2, Figure 3).

**Figure 2:**
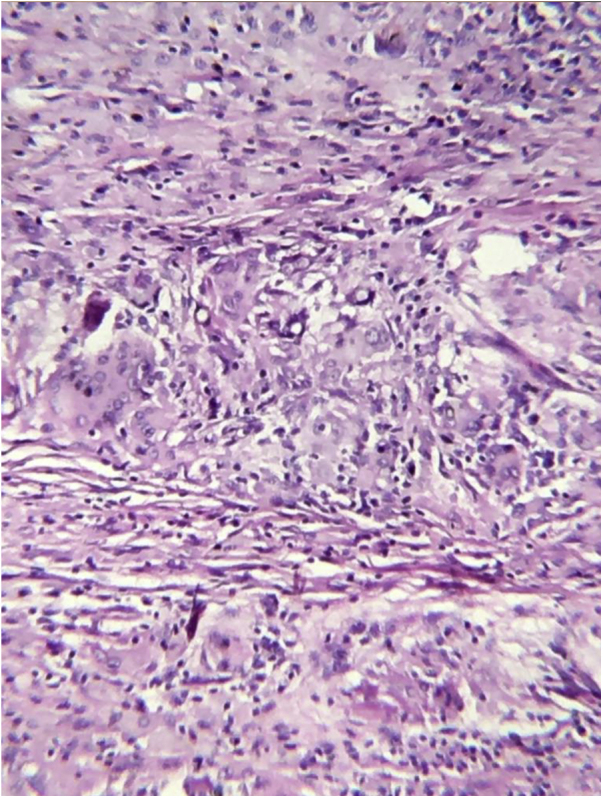
Histopathology of the surgical specimen. A granulomatous inflammatory reaction with a large number of giant cells containing rounded fungal elements can be seen (Hematoxylin & eosin, ×200).

**Figure 3:**
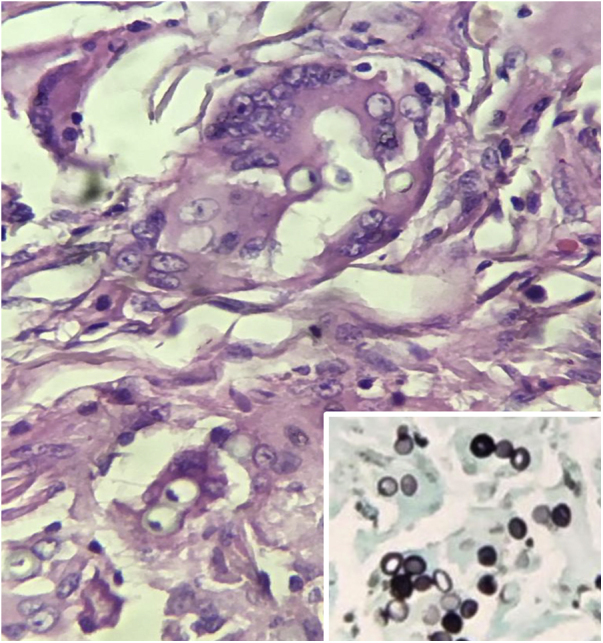
Histopathology of the surgical specimen. Presence of fungal elements, of similar size, with thick and birefringent walls inside giant cells (Hematoxylin & eosin, ×400). Microphotography: Rounded, birefringent fungal structures in a catenulate arrangement. (Grocott, ×600).

Surgical excision of the lesion was performed (Fig. 4) and itraconazole, at a dose of 100 mg, every 12 hours, orally, for six months was prescribed, in an attempt to prevent recurrence. The patient is in the eighth month of follow-up, progressing satisfactorily, without recurrence of the lesion.

**Figure 4:**
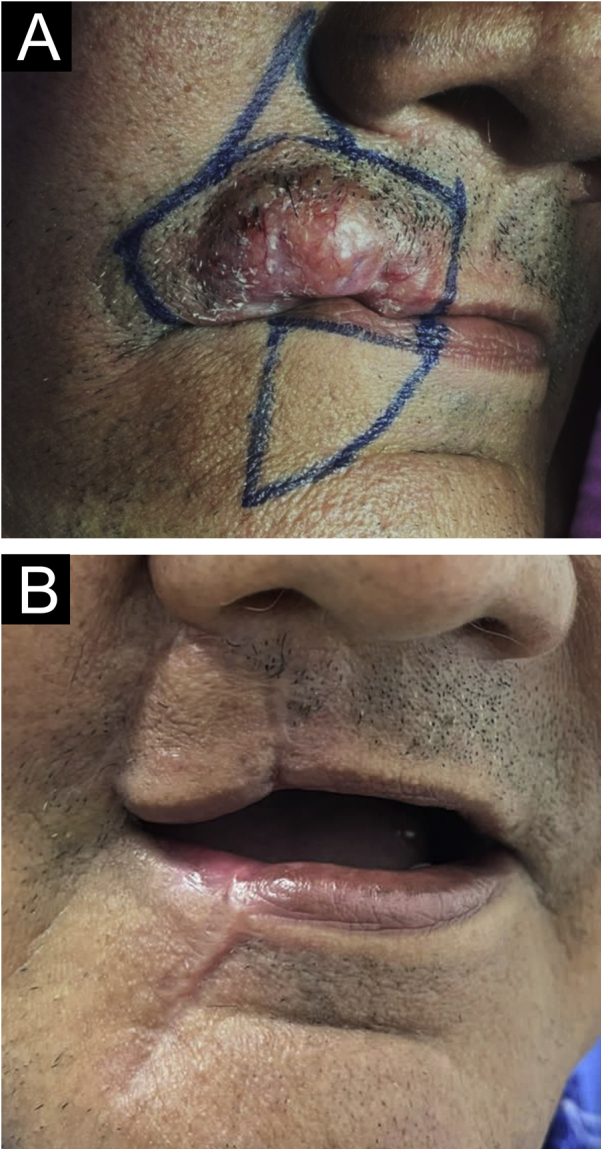
(A) Surgical markings before resection. (B) Five months after the procedure.

Lobomycosis (Jorge Lobo's disease) was first described in 1931 by the dermatologist Jorge Oliveira Lobo. It is a subcutaneous mycosis, usually characterized by nodular lesions with a keloid appearance; however, there may be lesion polymorphism, as plaques, papules, macules, verrucous lesions, ulcerations and scarring lesions; it evolutes slowly, sometimes making clinical diagnosis difficult.^1^, ^2^ The lesions are generally asymptomatic, although pruritus and dysesthesia may occur.^2^ The disease is caused by a yeast-like fungus called *Lacazia loboi*, which was recently renamed *Paracoccidioides lobogeorgii* following current taxonomic rules, after a broad nomenclature review.^3^

Although the disease occurs throughout Central and South America, it is mainly observed in the Amazon region, in patients from rural areas.^2^ The transmission mechanism is not exactly known, although traumatic implantation of the fungus into the skin is plausible. To date, the etiological agent has not been cultivated.

In most cases, lobomycosis is located mainly in the distal extremities and ears. Lip location is rare ‒ there are only two cases recorded in the consulted literature.^4^

The diagnosis is based on clinical aspects, direct mycological examination by scarification, scraping, or curettage of the lesion, and histopathology.^1^

Currently, there is no completely satisfactory therapy. The treatment of choice for unifocal and localized forms is surgical excision, with safety margins, associated or not with clinical treatment to prevent recurrence. Multifocal forms should be treated, whenever possible, with a combination of excision surgery and adjuvant systemic treatment. Effective medications previously reported in the literature include posaconazole, itraconazole and clofazimine. It is worth highlighting the need for long-term follow-up, as recurrence is possible.^2^, ^5^

New studies investigating the etiopathogenesis, transmission and treatment of lobomycosis are necessary to better elucidate this neglected and still obscure tropical disease that remains a challenge in dermatological practice.

## Financial support

None declared.

## Authors' contributions

Kananda Kesye Sousa Nunes: Drafting and editing of the manuscript; critical review of the literature; critical review of the manuscript; approval of the final version of the manuscript.

Antonio Pedro Mendes Schettini: Drafting and editing of the manuscript; effective participation in research orientation; critical review of the literature; critical review of the manuscript; approval of the final version of the manuscript.

Carlos Alberto Chirano Rodrigues: Effective participation in research orientation; intellectual participation in the propaedeutic and/or therapeutic conduct of the studied cases; critical review of the manuscript; approval of the final version of the manuscript.

Sinésio Talhari: Drafting and editing of the manuscript; effective participation in research orientation; critical review of the literature; critical review of the manuscript; approval of the final version of the manuscript.

## Conflicts of interest

None declared.
